# Evaluation the inhibitory effect of nicardipine on the metabolism of quetiapine

**DOI:** 10.3389/fphar.2025.1567044

**Published:** 2025-09-23

**Authors:** Jinzhao Yang, Hailun Xia, Qingqing Li, Ruibin Li, Lu Cao, Congrong Tang

**Affiliations:** ^1^ Department of Pharmacy, The Wenzhou Third Clinical Institute Affiliated to Wenzhou Medical University, Wenzhou, Zhejiang, China; ^2^ Department of Pharmacy, The First Affiliated Hospital of Wenzhou Medical University, Wenzhou, Zhejiang, China; ^3^ Ningbo Municipal Hospital of Traditional Chinese Medicine (TCM), Affiliated Hospital of Zhejiang Chinese Medical University, Ningbo, Zhejiang, China

**Keywords:** quetiapine, nicardipine, drug-drug interactions, UPLC-MS/MS, microsome

## Abstract

The aim of this study was to investigate the impact of calcium channel blockers (CCBs) and antihypertensive traditional Chinese medicine (TCM) on the metabolism of quetiapine. *In vitro*, two incubation systems of rat liver microsomes (RLM) and human liver microsomes (HLM) were established and optimized to explore potential interactions between five kinds CCBs (nicardipine, dilthiazem, lercanidipine, nimodipine, nitrendipine), five kinds antihypertensive TCM (quercetin, fangchinoline, apigenin, tetrandrine, and berberine) and quetiapine, and to evaluate their underlying inhibition mechanisms. *In vivo*, Sprague-Dawley rats were used to assess the interaction between quetiapine and nicardipine. The results showed that nicardipine had the highest inhibition rate (79.22%) against quetiapine metabolism among those drugs screened. The half-maximal inhibitory concentration (IC_50_) values for the inhibition of quetiapine metabolism by nicardipine in RLM and HLM were similar, at 10.29 ± 0.06 μM and 13.23 ± 0.37 μM, respectively. In RLM, nicardipine exhibited a mixed mechanism of competitive and non-competitive inhibition, while in HLM, it displayed a non-competitive and un-competitive inhibition mechanism. *In vivo* results indicated that nicardipine could significantly increase the main pharmacokinetic parameters AUC_(0-t)_, ${\text{AUC}}_{\left(0-\infty \right)}$ , and C_max_ of quetiapine, but decrease the AUC_(0-t)_ of its metabolite N-desalkylquetiapine. The findings of this study suggested that nicardipine had inhibited the metabolism of quetiapine, suggesting the dose adjustment or therapeutic drug monitoring of quetiapine should be conducted to achieve individualized therapy.

## 1 Introduction

At present, mental disorders including bipolar disorder, major depressive disorder, and schizophrenia, remain a leading cause of the global disease burden (Collaborators, 2022). Second-generation antipsychotic drugs (SGAs) constitute a novel class of antipsychotic medications utilized in the treatment of mental illnesses such as bipolar disorder and schizophrenia (Krejci et al., 2024).

Quetiapine, a dibenzothiazepine derivative, is classified as a SGA (Maneeton et al., 2016). It was approved by the U.S. Food and Drug Administration in 1997 for the treatment of schizophrenia, bipolar disorder, and depressive episodes (Han et al., 2024). Quetiapine has been a widely used antipsychotic for more than 30 years, and it has good efficacy in treating depressive episodes associated with bipolar disorder and major depressive disorder (Bakken et al., 2011; Sarris et al., 2022). Additionally, quetiapine demonstrates greater clinical efficacy as a first-line augmentation option for alleviating symptoms of treatment-resistant depression in the long-term management of this condition (Cleare et al., 2025). In a recent clinical study, when quetiapine was used in the treatment of refractory depression, the incidence of adverse reactions was 78.0% and the incidence of serious adverse reactions was 5.7% (Reif et al., 2023). Response and remission rates were highest for quetiapine in 6- or 8-week monotherapy, but the likelihood of discontinuation due to adverse events was higher (Li S. et al., 2024). In outpatient prescriptions, the combination of escitalopram and quetiapine is a common potential drug interaction (Chen and Ding, 2023). A clinical study demonstrated that the plasma concentration of quetiapine is dose-dependently affected by lamotrigine, suggesting the need for therapeutic drug monitoring of quetiapine (Hole et al., 2024). Quetiapine undergoes complete oral absorption and is primarily metabolized by cytochrome P450 (CYP) 3A4 into various metabolites (Grimm et al., 2006; Zubiaur et al., 2021), among which N-desalkylquetiapine is the principal active metabolite of quetiapine (Jensen et al., 2008). Recent studies indicate a potential for drug-drug interactions (DDI) between phenacetin and the CYP3A4 substrate quetiapine (Watermeyer et al., 2024). When quetiapine is used in combination with methadone, the plasma concentration levels of quetiapine metabolites increase (Heisel et al., 2024). Therefore, it is necessary to conduct essential drug-drug interactions studies, particularly for drugs that have not yet been reported and possess the potential for drug interactions.

Hypertension is prevalent in patients with bipolar disorder and anxiety (Johannessen et al., 2006), primarily due to patients with hypertension are more likely to experience negative emotions such as anxiety, stress, and depression (Kretchy et al., 2014). Consequently, there exists a significant possibility of antihypertensive drugs co-administrated with quetiapine. Harrison PJ, et al. have shown that calcium channel blockers (CCBs) targeting the L-type voltage-gated calcium channels play an essential role in the fundamental neuronal processes related to mental illnesses, and CCBs can also reduce psychiatric hospitalization and self-harm in patients with bipolar disorder (Hayes et al., 2019; Lintunen et al., 2022). Furthermore, attention to drug interactions mediated by natural products is increasing. However, there are few reports on the interactions between quetiapine and CCBs or traditional Chinese medicine (TCM).

**FIGURE 1:**
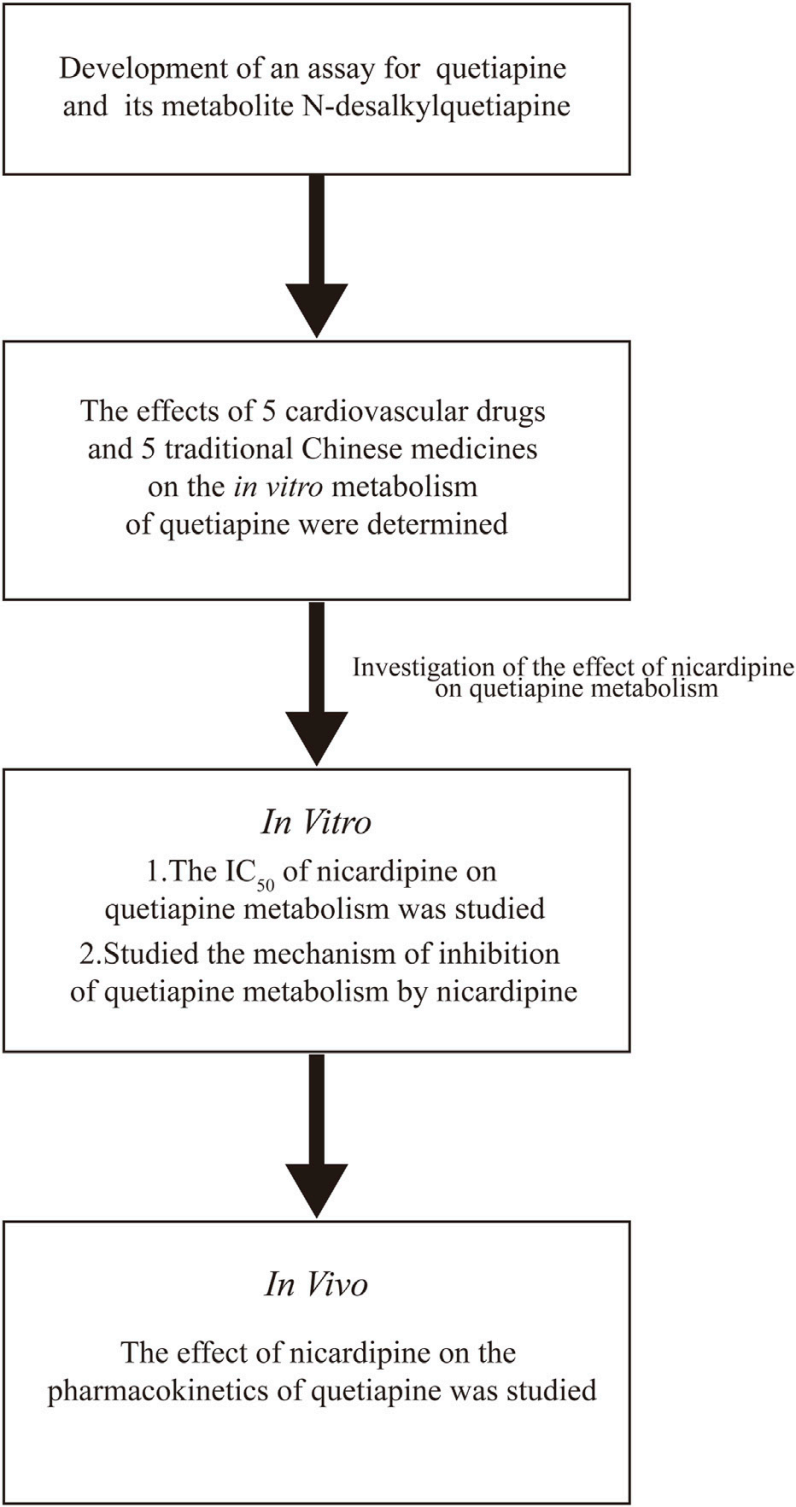
Schematic representation of the experimental method.

First, we developed a rapid, sensitive, and accurate UPLC-MS/MS method for detecting the concentration of quetiapine and its metabolite, N-desalkylquetiapine, based on previous studies (Fisher et al., 2012; Pan et al., 2012; Xiong et al., 2013; Li et al., 2017; Luo et al., 2023). In this study, we selected five kinds L-type voltage-gated calcium channel CCBs and five kinds TCM with antihypertension to explore their interactions with quetiapine. Initially, *in vitro,* we utilized rat liver microsomes (RLM) and human liver microsomes (HLM) to investigate their effects on the metabolism of quetiapine and their underlying mechanisms. Subsequently, *in vivo*, based on the results of the *in vitro* experiments, we employed Sprague-Dawley rats to examine the impact of nicardipine on the pharmacokinetic parameters of quetiapine. The schematic of the experiment method is shown in Figure 1. We expected to provide some data support for the individualized treatment of quetiapine in clinical practice.

## 2 Materials and methods

### 2.1 Chemicals and reagents

Quetiapine, N-desalkylquetiapine and lurasidone (internal standard, IS) were obtained from Shanghai Chuangsai Technology Co., Ltd. (Shanghai, China). Ten kinds drugs (nicardipine, dilthiazem, lercanidipine, nimodipine, nitrendipine, quercetin, fangchinoline, apigenin, tetrandrine, and berberine) were also got from Shanghai Chuangsai Technology Co., Ltd. (Shanghai, China). The RLM and HLM were purchased from iPhase Pharmaceutical Services Co., Ltd. (Jiangsu, China). Reduced nicotinamide adenine dinucleotide phosphate (NADPH) was procured from Shanghai Aladdin Biochemical Technology Co., Ltd. (Shanghai, China). Methanol and acetonitrile were provided by Merck Company (Darmstadt, Germany).

### 2.2 UPLC-MS/MS condition

The chromatographic separation of quetiapine, N-desalkylquetiapine, and IS was achieved utilizing an ultra performance liquid chromatography tandem mass spectrometry (UPLC-MS/MS) equipped with a Waters Acquity BEH C18 chromatographic column (2.1 mm × 50 mm, 1.7 μm; Milford, MA, United States). At a column temperature of 40 °C, the mobile phase, composed of 0.1% formic acid (mobile phase A) and acetonitrile (mobile phase B), was eluted at a gradient flow rate of 0.4 mL/min for a duration of 2.0 min. Mass spectrometry information was obtained using a Waters Xevo TQS triple quadrupole tandem mass spectrometer equipped with an electrospray ionization (ESI) source (Milford, MA, United States), operating in positive mode with multiple reaction monitoring (MRM). The ion transitions for quetiapine, N-desalkylquetiapine, and IS were *m/z* 384.04 → 252.95, m*/z* 295.99 → 209.93, and *m/z* 493.05 → 165.99, respectively.

### 2.3 Incubation condition

200 μL enzymatic kinetic incubation system was adjusted according to the measurement purpose with the corresponding incubation conditions. The determination of enzymatic kinetic parameters of quetiapine in RLM and HLM were performed under the following conditions: 1–200 μM quetiapine, 0.3 mg/mL RLM or HLM, 0.1 M PBS buffer, 1 mmol/L NADPH, with triplicate samples for each concentration. The mixture without NADPH was pre-incubated at 37 °C in a water bath for 5 min, then 10 μL NADPH (20 mM) was added to initiate the reaction. Following a further 30 min incubation, the reaction was terminated by freezing at −80 °C. Prior to sample thawing, 400 μL acetonitrile was added to precipitate proteins, and 20 μL lurasidone (200 ng/mL) was added as IS. After complete dissolution of the samples, they were vortexed for 2 min and centrifuged for 10 min (13,000 rpm), then the supernatants were taken for UPLC-MS/MS detection and analysis.

The potential interactions between quetiapine and five kinds L-type voltage-gated CCBs (nicardipine, dilthiazem, lercanidipine, nimodipine, nitrendipine) and five kinds of TCM (quercetin, fangchinoline, apigenin, tetrandrine, and berberine) with antihypertension were investigated. The 200 μL incubation system was comprised of PBS buffer (0.1 M), RLM or HLM (0.3 mg/mL), NADPH (1 mmol/L), quetiapine and 10 kinds drugs (100 μM). There is a high potential for drug-drug interactions between these ten drugs that could have effects on the metabolic process of quetiapine. The concentration of quetiapine is based on the Michaelis-Menten constant (K_m_).

Subsequently, the half-maximal inhibitory concentration (IC_50_) of nicardipine on quetiapine in RLM or HLM was determined, with nicardipine concentrations set at: 0, 0.01, 0.1, 1, 10, 25, 50, and 100 μM. The concentration of quetiapine based on K_m_ was 48.79 μM in RLM and 50.80 μM in HLM, respectively. Finally, the potential mechanism type of inhibition of quetiapine metabolism by nicardipine was further examined. The concentration of quetiapine was set according to the corresponding K_m_ values, at 12.20, 24.40, 48.79, 97.58 μM in RLM, and 12.70, 25.40, 50.80, 101.60 μM in HLM, respectively. Moreover, the concentration of nicardipine was set according to the corresponding IC_50_ values, at 0, 2.57, 5.15, 10.29 μM in RLM, and 0, 6.62, 13.23, 26.46 μM in HLM, respectively. Sample processing was as described above.

### 2.4 Pharmacokinetics research

Due to the similarity between their hepatic enzyme system and humans, Sprague-Dawley male rats are widely used in pharmacokinetics (Martignoni et al., 2006; Riccardi et al., 2018). This pharmacokinetic study was conducted on eight healthy male Sprague-Dawley rats provided by the Animal Experiment Center of the First Affiliated Hospital of Wenzhou Medical University (Zhejiang, China). Animals were housed in an environment with a temperature of 20 °C–26 °C, relative humidity of 55% ± 15%, and a light-dark cycle of 12 h/day, in strict following the guidelines for the care and use of laboratory animals by the National Research Council. The research protocol was adhered to the ARRIVE guidelines and was approved by the Experimental Animal Ethics Committee of the First Affiliated Hospital of Wenzhou Medical University (WYYY-IACUC-AEC-2023–064). Before the formal experiment, in order to mitigate the influence of food on drug absorption, Sprague-Dawley rats were fasted for 12 h and then randomly divided into two groups. Nicardipine and quetiapine are dissolved using corn oil. In the co-administration group (n = 4), each rat was gavaged with 4 mg/kg nicardipine, whereas in the control group (n = 4), each rat was given an equal amount of corn oil orally to reduce solvent interference. 30 min later, both groups of Sprague-Dawley rats were given 15 mg/kg quetiapine by gavage. Subsequently, blood samples were collected from the rat’s tail vein into heparinized centrifuge tubes at 15 min, 30 min, 45 min, and 1, 1.5, 2, 3, 4, 6, 8, 12, 24 h post-quetiapine administration. Following centrifugation at 8,000 rpm for 10 min, 100 μL of plasma supernatant was precisely taken into a new clean and dry 1.5 mL EP tube, where 300 μL acetonitrile and 10 μL IS working solution (200 ng/mL) were added, and after vortexing and centrifugation, the supernatants were taken for injection and analysis.

### 2.5 Statistical analysis

The Michaelis-Menten curves, IC_50_ curves, Lineweaver–Burk plots, and the mean plasma concentration–time curves were generated using GraphPad Prism software (version 9.5; GraphPad Software Inc., San Diego, CA). The pharmacokinetic parameters were derived using the non-compartmental model analysis with Drug and Statistics software (version 3.0 software, Mathematical Pharmacology Professional Committee of China, Shanghai, China). Statistical differences between the co-administered group and the control group were analyzed using the *t*-test of SPSS (version 24.0; SPSS Inc., Chicago, IL, USA), with *P < 0.05* considered statistically significant.

## 3 Results

### 3.1 Chromatographic characterization and mass spectrometric information of analytes

**FIGURE 2:**
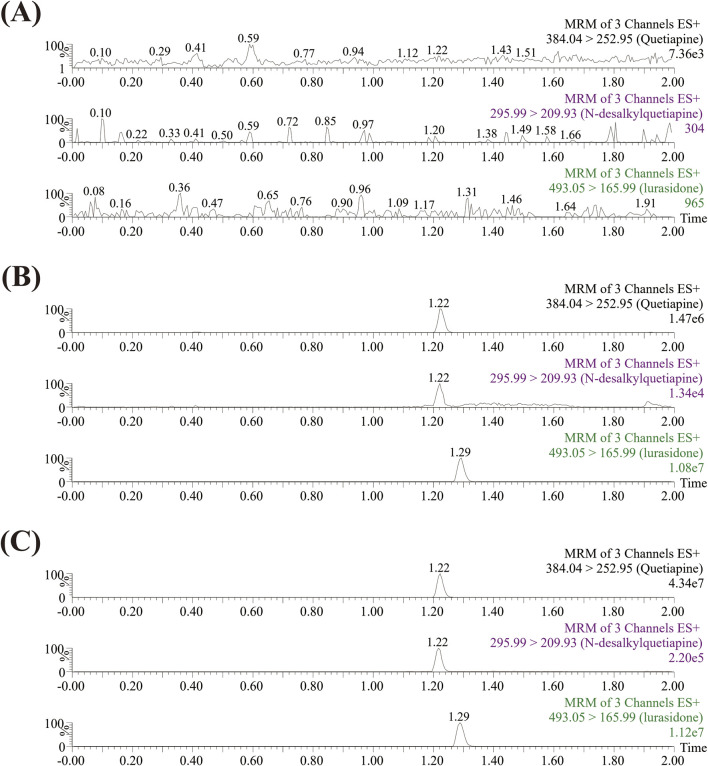
Typical UPLC-MS/MS chromatograms of quetiapine, N-desalkylquetiapine, and IS. Blank rat plasma **(A)**; Blank rat plasma with 1 ng/mL standards **(B)**; Rat plasma sample from the formal animal experiment **(C)**.

The UPLC-MS/MS analytical method was developed according to the Guideline on bioanalytical method validation published by EMA (EMA, 2015). It could be seen from Figure 2 that the analytes were well separated within an elution time of 2.0 min, and there was no interference from endogenous substances. The retention times of quetiapine and its main metabolite (N-desalkylquetiapine), and IS were 1.22 min, 1.22 min, and 1.29 min, respectively. Quetiapine and N-desalkylquetiapine exhibited good linearity within the range of 0.1–100 ng/mL and 0.1–500 ng/mL, respectively. The linear regression equation for quetiapine was y = 0.101313x + 0.011457, *r* ^2^ = 0.9946, and for N-desalkylquetiapine was y = 0.000869966x + 0.0000589335, r2 = 0.9987, respectively. Additionally, the lower limit of quantification (LLOQ) of quetiapine and N-desalkylquetiapine both were 0.1 ng/mL.

### 3.2 Nicardipine had the strongest suppression strength on quetiapine metabolism among the 10 kinds drugs

**FIGURE 3:**
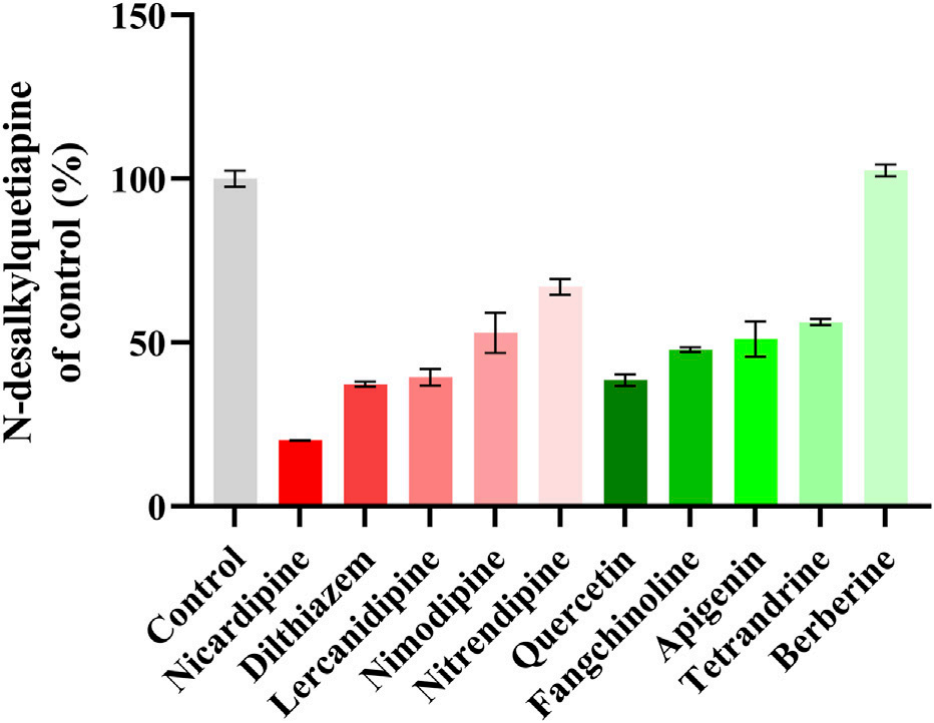
A comparison of the inhibitory effects on the formation of N-desalkylquetiapine by five kinds L-type voltage-gated CCBs and five kinds antihypertensive TCM in RLM. Data are expressed as the mean ± standard deviation (SD). N = 2.

Figure 3 shows that nicardipine was the drug that most inhibited the metabolism of quetiapine among the five kinds L-type voltage-gated CCBs, and the strongest inhibitory ability among the five kinds antihypertensive TCM was quercetin. However, the inhibition rate of nicardipine was 79.22%, which was still higher than that of quercetin (61.47%). The results indicated that nicardipine had the strongest inhibitory effect on quetiapine metabolism among the 10 screened drugs.

### 3.3 Nicardipine inhibited the metabolism of quetiapine by a mixed mechanism in both RLM and HLM

**FIGURE 4:**
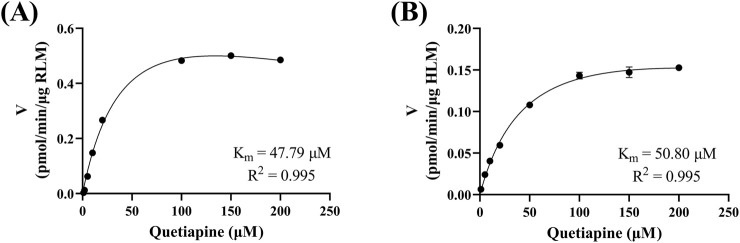
Michaelis-Menten curves of quetiapine in RLM **(A)** and HLM **(B)**. Data are expressed as the mean ± SD, n = 3.

**FIGURE 5:**
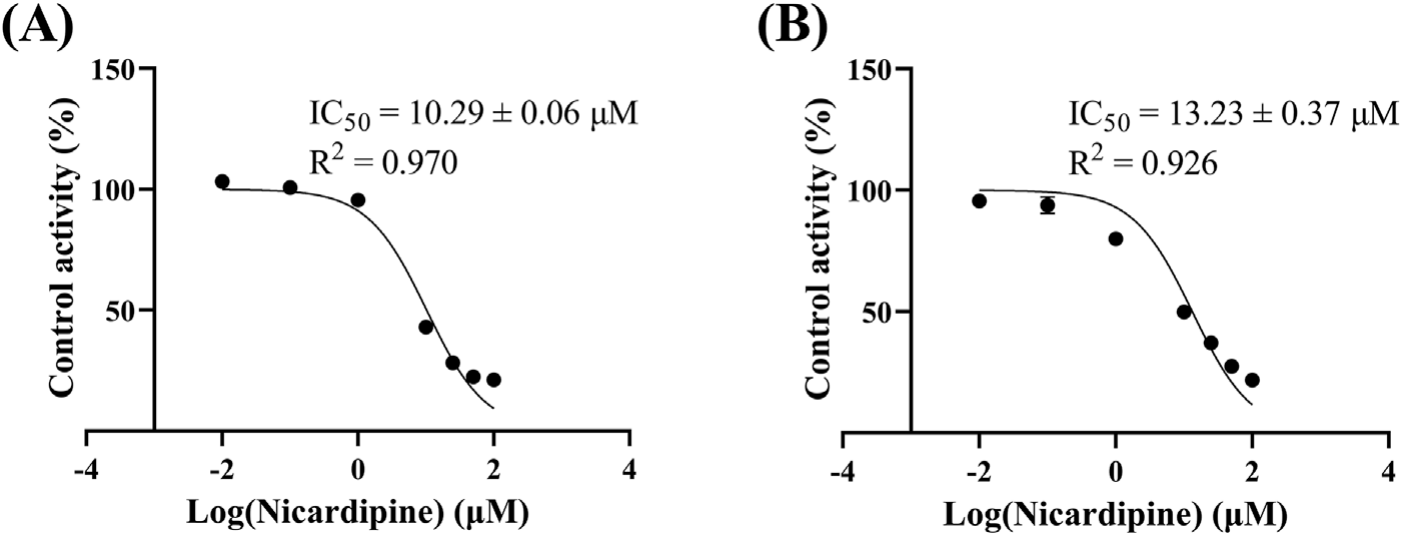
IC_50_ curves of nicardipine at different concentrations (0.01, 0.1, 1, 10, 25, 50, and 100 μM) for the inhibition of quetiapine in RLM **(A)** and HLM **(B)**. Data are expressed as the mean ± SD, n = 3.

**FIGURE 6:**
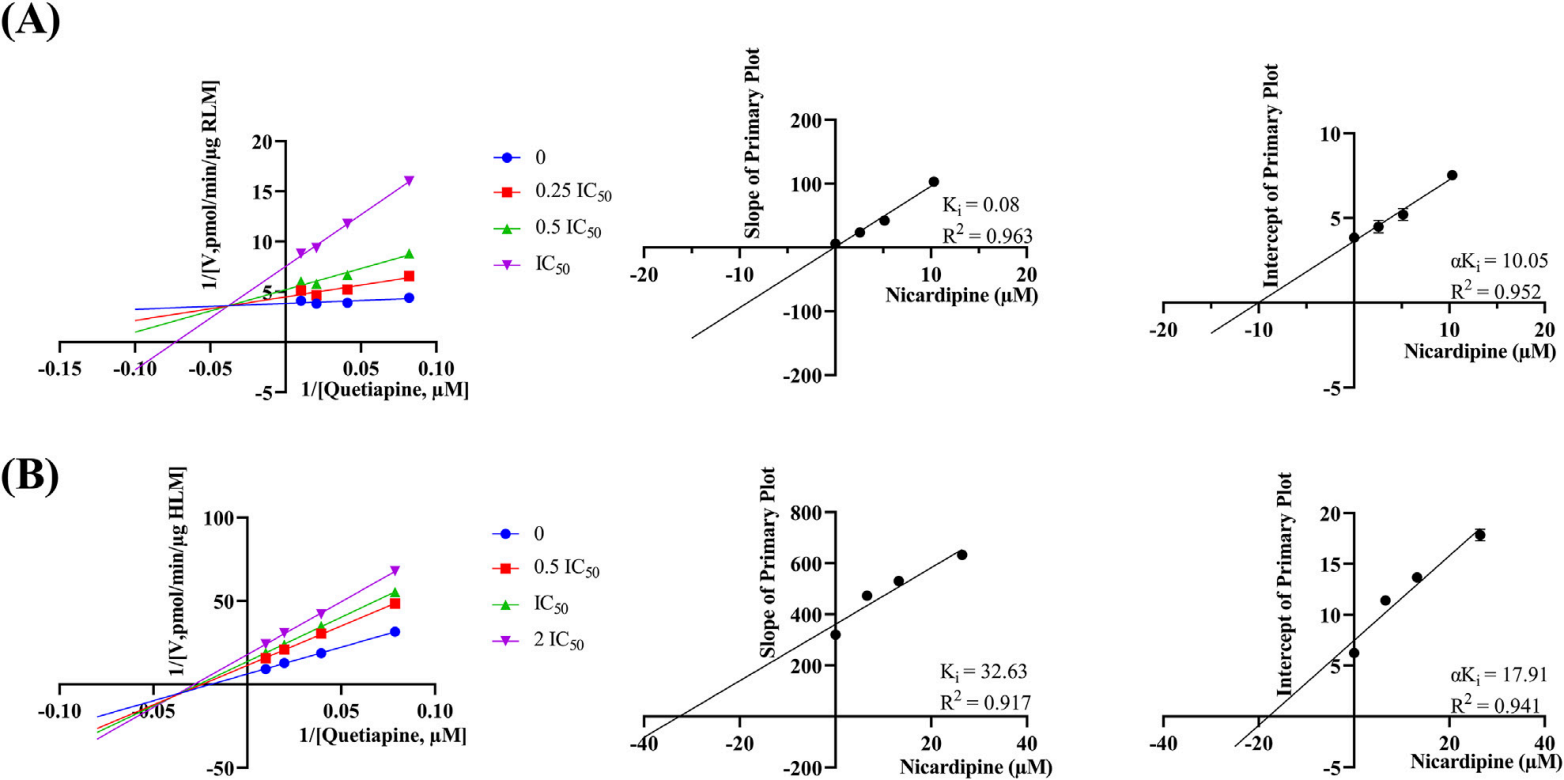
Lineweaver-Burk plots, K_i_ secondary plots, and αK_i_ secondary plots of nicardipine at different concentrations inhibiting quetiapine metabolism in RLM **(A)** and HLM **(B)**. Data are expressed as the mean ± SD, n = 3.

Figure 4 reveals that the K_m_ of quetiapine in RLM and HLM were close, at 48.79 μM and 50.80 μM, respectively, suggesting that the activity of RLM in catalyzing the metabolism of quetiapine may be similar to that of HLM. K_m_ is the concentration of the substrate when the enzymatic reaction reaches half of the maximum speed. It is a characteristic physical quantity of the enzyme and its magnitude is related to the nature of the enzyme. Using K_m_ as the substrate concentration, the effect of other factors on the reaction rate was clearly observed. According to Figure 5, the IC_50_ of nicardipine inhibiting quetiapine metabolism in RLM and HLM was also not significantly different, with IC_50_ values of 10.29 ± 0.06 μM and 13.23 ± 0.37 μM, respectively. Interestingly, Figure 6 indicates that nicardipine inhibited quetiapine metabolism in both RLM and HLM through a mixed mechanism type. In RLM, nicardipine exhibited a mixed mechanism combining competition and non-competition, with K_i_ = 0.08 μM and αK_i_ = 10.05 μM, while in HLM, it showed a combination of non-competition and un-competition, with K_i_ = 32.63 μM and αK_i_ = 17.91 μM.

### 3.4 Nicardipine enhanced the drug exposure of quetiapine in Sprague-Dawley rats

**FIGURE 7:**
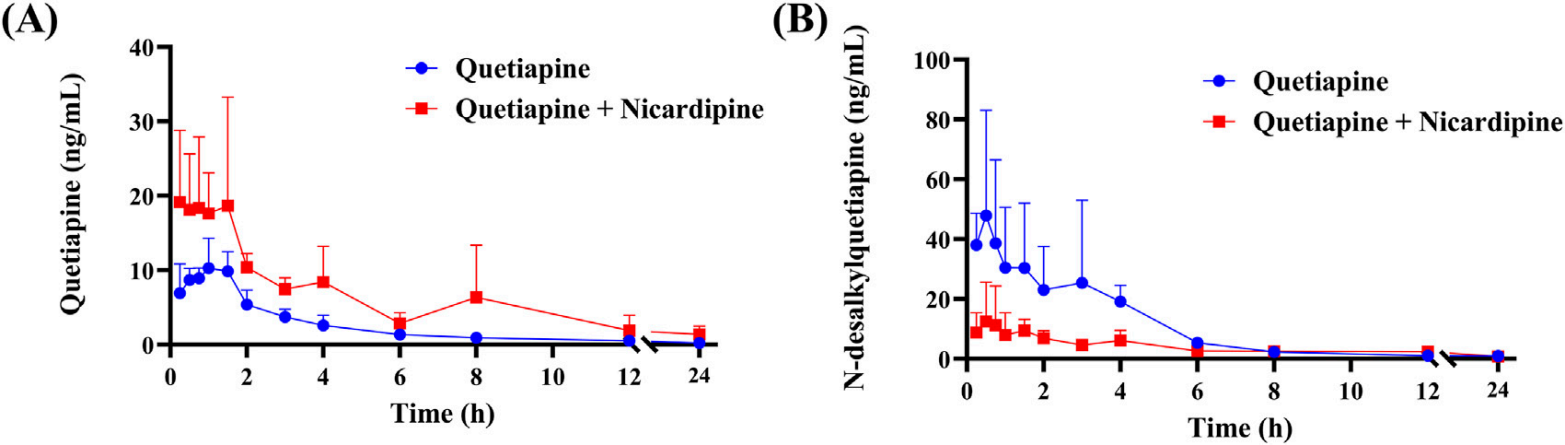
Mean plasma concentration-time curve of quetiapine **(A)** and N-desalkylquetiapine **(B)** in two groups of rats. Data are presented as the means ± SD, n = 4.

The mean plasma concentration-time curves of quetiapine and its main metabolite (N-desalkylquetiapine) are shown in Figure 7. The results indicated that the co-administration of nicardipine not only increased the drug exposure to quetiapine in Sprague-Dawley rats, but also reduced the production of N-desalkylquetiapine. According to the pharmacokinetic parameters in Table 1, nicardipine increased the AUC_(0-t)_, ${\text{AUC}}_{\left(0-\infty \right)}$ , and C_max_ of quetiapine by 1.94-, 1.85-, and 1.38-fold, respectively, and reduced the elimination rate of quetiapine by 63.40%. However, nicardipine decreased the AUC_(0-t)_ of N-desalkylquetiapine by 56.30% (Table 2). Therefore, all the above data indicated that nicardipine had inhibitory effect on the metabolism of quetiapine in rats.

**TABLE 1:** Main pharmacokinetic parameters of quetiapine in the two groups of Sprague-Dawley rats (n = 4, mean ± SD).

Parameters	Quetiapine	Quetiapine + nicardipine
AUC_(0-t)_ (ng/mL*h)	36.05 ± 5.48	105.95 ± 30.32*
${\text{AUC}}_{\left(0-\infty \right)}$ (ng/mL*h)	38.07 ± 6.91	108.32 ± 31.35*
t_1/2_ (h)	5.12 ± 3.56	5.16 ± 2.82
T_max_ (h)	0.94 ± 0.52	0.88 ± 0.52
CL_z/F_ (L/h/kg)	403.52 ± 70.45	147.70 ± 42.64**
C_max_ (ng/mL)	12.02 ± 2.28	28.77 ± 10.73*

*P < 0.05, **P < 0.01, compared with quetiapine alone. AUC, area under the plasma concentration-time curve; t_1/2_, elimination half time; T_max_, peak time; CL_z/F_, plasma clearance; C_max_, maximum plasma concentration.

**TABLE 2:** Main pharmacokinetic parameters of N-desalkylquetiapine in the two groups of Sprague-Dawley rats (n = 4, mean ± SD).

Parameters	Quetiapine	Quetiapine + nicardipine
AUC_(0-t)_ (ng/mL*h)	160.62 ± 63.16	70.19 ± 22.75*
${\text{AUC}}_{\left(0-\infty \right)}$ (ng/mL*h)	161.47 ± 62.64	80.29 ± 27.62
t_1/2_ (h)	3.19 ± 1.76	8.54 ± 5.49
T_max_ (h)	0.31 ± 0.13	0.69 ± 0.55
CL_z/F_ (L/h/kg)	101.52 ± 30.08	209.99 ± 92.45
C_max_ (ng/mL)	50.66 ± 32.77	14.98 ± 11.59

*P < 0.05, compared with quetiapine alone. AUC, area under the plasma concentration-time curve; t_1/2_, elimination half time; T_max_, peak time; CL_z/F_, plasma clearance; C_max_, maximum plasma concentration.

## 4 Discussion

Atypical antipsychotics, also known as SAGs, include clozapine, olanzapine, risperidone, quetiapine, aripiprazole, and ziprasidone, which have the advantages of a broad spectrum of action, good efficacy, and a good safety profile compared with traditional antipsychotics, and can improve the quality of life of patients (Mauri et al., 2018). They are now considered as the first-line treatments for schizophrenia (Hamsa et al., 2023). Quetiapine primarily exerts its pharmacological activity by binding to dopamine D_2_ and serotonin (5-HT) receptors(Mauri et al., 2018). Studies have shown that quetiapine has similar efficacy to first-generation antipsychotics, but it does not increase the level of hyperprolactinemia and related somatic severe adverse reactions (Krøigaard et al., 2022). Thus, it serves as an excellent alternative for patients sensitive to other antipsychotic drugs like clozapine or olanzapine. However, a study indicates that potent and moderate CYP3A4 inducers can increase the apparent clearance of quetiapine by approximately fourfold, while potent CYP3A4 inhibitors reduce it by 93%. DDI can affect the elimination of quetiapine in the body, and conducting relevant research can reduce the risk of potential DDI occurring.

It has been observed that patients with bipolar disorder or schizophrenia have higher cardiovascular morbidity and mortality, partly due to an increased risk of hypertension or poor treatment outcomes, with an overall incidence ratio of hypertension of 1.27 (Ayerbe et al., 2018). One study reported a 39% prevalence of hypertension in schizophrenia and related disorders(Sudarshan and Cheung, 2023). Consequently, there is a high probability that patients with mental disorders would co-administration of quetiapine with antihypertensive medications. It is also known that CCBs targeting the L-type voltage-gated calcium channels play a key role in the fundamental neuronal processes associated with mental illnesses (Harrison et al., 2022). Moreover, there is a growing preference for using natural products as dietary supplements, food additives, and medications to enhance health. Thus, this study systematically screened five kinds CCBs targeting the L-type voltage-gated calcium channels and five kinds antihypertensive TCM to explore their impact on quetiapine metabolism.

Based on previous literature, we have developed a UPLC-MS/MS analytical method to rapidly and sensitively determine the concentration of quetiapine and its metabolite N-desalkylquetiapine. Quetiapine and its metabolite N-desalkylquetiapine have good linearity. Based on this analytical method, we quantified quetiapine and N-desalkylquetiapine *in vitro* and *in vivo* to study the effect of nicardipine on quetiapine metabolism.

*In vitro* results indicated that nicardipine inhibited 79.22% of quetiapine metabolism, making it to be the most potent inhibitor among the 10 drugs. Previous studies have found that nicardipine can significantly affect drugs metabolized via CYP3A4, resulting in slower metabolism and increased drug exposure (Xia et al., 2012; Liu et al., 2023; Chen et al., 2024). Its IC_50_ values for quetiapine metabolism in RLM and HLM were 10.29 ± 0.06 μM and 13.23 ± 0.37 μM, respectively. It has been reported that nicardipine is a relatively potent inhibitor of human CYP3A4 (Nakamura et al., 2005). Additionally, research by Ya-nan Liu, et al. has shown that nicardipine can significantly inhibit the metabolism of alectinib, a CYP3A4 substrate, both *in vitro* and *in vivo* (Liu et al., 2023). Quetiapine is extensively metabolized in the liver by the CYP450 system, primarily via CYP3A4. Therefore, nicardipine may reduce the metabolic rate of quetiapine *in vitro* by inhibiting CYP3A4 activity. In terms of the inhibition mechanism, in RLM, nicardipine inhibited quetiapine metabolism through a mixed mechanism of competition and non-competition, with K_i_ = 0.08 μM and αK_i_ = 10.05 μM, and exhibited a non-competitive and uncompetitive inhibition mechanism in HLM, with K_i_ = 32.63 μM and αK_i_ = 17.91 μM. Competitive inhibition occurs when the inhibitor competes with the substrate for the enzyme’s active site. Non-competitive inhibition occurs when the inhibitor binds to an allosteric site, altering the enzyme’s activity regardless of substrate binding. Uncompetitive inhibition occurs when the inhibitor binds only to the enzyme-substrate complex, stabilizing it and preventing the reaction. Mixed inhibition is a combination, where the inhibitor can bind both to the free enzyme and the enzyme-substrate complex, affecting enzyme activity in multiple ways (Zhou et al., 2007; Kamel and Harriman, 2013; Ye et al., 2024). K_i_ reflects the inhibitory strength of the inhibitor against the target, thus suggesting that nicardipine may have a stronger inhibitory effect on quetiapine metabolism in RLM than in HLM. The human liver primarily expresses CYP3A4/5, while the rat liver expresses CYP3A1/2. These isoforms share ∼70–85% sequence identity but differ in substrate/inhibitor binding patterns (Fujino et al., 2021). The rat CYP3A isoforms often show lower abundance (<1 fmol/μg), compared to 30–90 fmol/μg for human CYP3A4 (Hammer et al., 2021). Human CYP3A4 exhibits a highly flexible and large (∼520 Å^3^) active site, enabling it to accommodate diverse ligands (Wright et al., 2019). By contrast, rat CYP3A1 enzyme have more restricted active sites with different key residues, which direct distinct ligand orientations (Handa et al., 2013). These differences may have led to different inhibitory mechanisms. Although RLM provide valuable mechanistic insights, the discrepancies emphasize the need for caution when interpreting animal data and underscore the importance of confirming inhibitory mechanism in HLM or clinical studies.

This pharmacokinetic study was conducted in a relatively small number of animals (4 rats per group). The limited number of subjects may reduce the statistical power of the analysis and restrict the generalizability of the findings. *In vivo* results in Sprague-Dawley rats showed that nicardipine significantly increased the AUC_(0-t)_, ${\text{AUC}}_{\left(0-\infty \right)}$ and C_max_ of quetiapine by 1.94-, 1.85- and 1.39-fold, respectively, increasing the exposure to quetiapine in rats. In addition, nicardipine significantly reduced the AUC_(0-t)_ of N-desalkylquetiapine, a metabolite of quetiapine, by 56.3%. Another study found that nicardipine significantly altered the *in vivo* metabolism of almonertinib, increasing its AUC_(0-t)_, ${\text{AUC}}_{\left(0-\infty \right)}$ and C_max_ by 0.95-, 0.87- and 1.01-fold, respectively (Chen et al., 2024). N-desalkylquetiapine is the main active metabolite of quetiapine metabolized by CYP3A4. Unlike the quetiapine, it effectively inhibits the norepinephrine transporter and partially activates the 5-HT_1A_ receptor, which is generally considered to be the cause of its antidepressant effects (Jensen et al., 2008). Thus, when nicardipine is used in combination with quetiapine, the rise in plasma exposure of quetiapine may increase the risk of adverse effects, such as hepatic impairment (Ko et al., 2023), acute pancreatitis (Li D. et al., 2024), oral ulcers (Srifuengfung et al., 2022), while the decrease in plasma exposure of N-desalkylquetiapine may reduce its antidepressant effects. When a combination of quetiapine and nicardipine must be used, it is necessary to adjust the administered dose of quetiapine and closely monitor the efficacy and adverse events to enhance the quality of life of the patient.

## 5 Conclusion

Quetiapine may be co-administered with antihypertensive drugs. Especially, nicardipine could cause fluctuations in quetiapine plasma concentration. Therefore, when quetiapine is used in combination with antihypertensive drugs in clinical practice, special attention should be paid to the potential drug-drug interactions with nicardipine. However, considering interspecies differences, further clinical studies are needed to clarify the possibility of drug interactions occurring in humans.

## Data Availability

The original contributions presented in the study are included in the article/supplementary material, further inquiries can be directed to the corresponding author.

## Abbreviations

CCBs, calcium channel blockers; TCM, traditional Chinese medicine; RLM, rat liver microsomes; HLM, human liver microsomes; IC50, half-maximal inhibitory concentration; SGA, Second-generation antipsychotic drug; CYP, cytochrome P450; IS, internal standard; NADPH, Reduced nicotinamide adenine dinucleotide phosphate; ESI, electrospray ionization; MRM, reaction monitoring; UPLC-MS/MS, ultra performance liquid chromatography tandem mass spectrometry; Km, Michaelis-Menten constant; SD, standard deviation; DDI, drug-drug interactions.
